# The Experience of Screening for HIV/AIDS Medical Studies among African-American/Black and Latino/Hispanic Persons Living with HIV/AIDS: A Mixed-Methods Exploration

**DOI:** 10.4172/2155-6113.1000223

**Published:** 2013-07-29

**Authors:** Stephanie Engel, Marya Viorst Gwadz, Charles M Cleland

**Affiliations:** 1 Karolinska University Hospital, Solna, SE-17176 Stockholm, Sweden; 2 New York University College of Nursing, New York, NY, USA

**Keywords:** HIV/AIDS medical studies, Clinical trials, Screening, Racial/ethnic disparities, African American, Latino

## Abstract

**Objective:**

African-American/Black and Latino/Hispanic persons living with HIV/AIDS (i.e., “PLHA of color”) are under-represented in HIV/AIDS medical studies (HAMS). A crucial entry point into HAMS is screening, but PLHA of color face serious barriers to screening compared to Whites. Recently we evaluated a social/behavioral intervention that substantially increased rates of HAMS screening among PLHA of color. Yet very little is known about the actual screening experience for these under-represented subgroups. Thus, the objectives of the present study were to explore participants’ motivations for and experiences of HAMS screening.

**Methods:**

A total of 186 participants in the larger study’s intervention arm were screened for HAMS, 35 of whom also participated in qualitative interviews. Participants engaged in a structured interview about the screening experience at 4- and 12- months post-baseline (14 items, Cronbach's α=0.72). Further, from a qualitative data set we purposively selected a set of three case studies to contextualize and enrich quantitative findings on screening experiences.

**Results:**

The screening experience was overwhelmingly positive. Almost all participants reported being treated with dignity and respect, did not feel they were being treated like a “guinea pig,” and experienced a high level of trust in the setting and the screener, with no gender or racial/ethnic differences, and no differences based on whether participants were found eligible for HAMS during screening. A number of areas where screening could be improved were also identified.

**Conclusions:**

Despite the complex barriers PLHA of color experience to screening for HAMS, the experience of screening was positive. Moreover, HAMS screening experiences were positive regardless of gender, race/ethnicity, or HAMS eligibility. HAMS screening can therefore be a productive learning experience that may reduce patient concerns about participating in HAMS. As such, fostering screening among PLHA of color can be an important component of reducing racial/ethnic disparities in HAMS.

## Introduction

HIV/AIDS medical studies (HAMS) are research studies designed to evaluate promising therapies to combat HIV infection; treat and prevent opportunistic infections and cancers associated with HIV/AIDS; address the complications of antiretroviral therapy; reconstitute immune systems damaged by HIV/AIDS; and better understand the biomedical consequences of HIV infection [1]. As such, HAMS are vital to the development of new treatment regimens for HIV infection. However, persons living with HIV (PLHA) from African-American/Black and Latino/Hispanic backgrounds in the United States, referred to here as “PLHA of color,” are involved in HAMS at disproportionally low rates, with African-Americans/Blacks experiencing the lowest rates of enrollment [2–4]. This disproportionality is problematic because it may limit the generalizability of HAMS findings, including to the populations most adversely affected by HIV [5,6]. Moreover, the under-representation of populations of color in HAMS denies members of these groups the opportunity to contribute to medical research and also prevents their access to the high level of care made available through HAMS, as well as the possibility of receiving new treatments for HIV [7–11].

### Description of the process of enrolling into HAMS

In the United States, HAMS are conducted by clinical trials research units (CTRUs), located in hospital centers and other medical settings. PLHA typically gain access to HAMS through a screening process used to determine eligibility. The screening process may be formal or informal and typically includes a health history interview, review of the characteristics of HAMS for which the participant may be eligible, medical testing if needed, review of consent forms, and coordination with the patient’s primary care provider. PLHA found eligible for HAMS during screening may then enroll into the trial or study. However, PLHA of color are substantially less likely to gain access to screening than their White peers [7, 12].

### Barriers to HAMS screening and enrollment

The set of barriers that reduce access to HAMS for PLHA of color are complex and operate at multiple levels of influence [8, 9]. At the level of individual PLHA, barriers include low levels of knowledge of HAMS and little awareness of how to access trials, along with strong feelings of fear and distrust of trials [13,14]. Yet, paradoxically, PLHA of color also report high levels of willingness to explore HAMS [8–10,15]. PLHA of color also experience social-level barriers to HAMS, as high rates of distrust and a history of exclusion appear to perpetuate social norms that discourage participation in medical research [12,16,17]. At the organizational level, PLHA of color are less likely to be referred to screening for HAMS by their health care providers compared to Whites [18]. Moreover, structural factors, such as difficulty navigating the unfamiliar CTRU setting and system, appear to impede their access to screening and enrollment [19,7].

### The ACT2 Intervention to increase screening for HAMS

We recently conducted a randomized controlled trial to evaluate the efficacy of a peer-driven intervention to increase HAMS screening rates for PLHA of color, called the “ACT2” intervention [8,10,20]. The multi-component intervention was comprised of small group and individual sessions (6 hours total structured activities), the opportunity to educate peers about HAMS, and navigation [21,22] to resolve barriers during the screening process [23]. The intervention focuses on fostering motivation to explore *screening*, a relatively low-risk and non-threatening activity, in contrast to the decision whether to enroll in HAMS, a potentially higher-risk activity. This strategy was a means of encouraging PLHA of color to explore HAMS and increase access to the CTRU setting. The ACT2 intervention was highly efficacious in increasing rates of screening for HAMS among PLHA of color, with approximately 50% of those in the intervention arm screened, compared to < 5% of controls [10]. Approximately half of those screened were found eligible for a study, and 9 out of 10 found eligible enrolled, primarily into observational biomedical studies [24].

#### The importance of screening

Screening is a crucial early step necessary to access HAMS, and also has potential to yield a number of other benefits whether or not an individual enrolls in a study. First, it communicates to CTRUs that PLHA of color are interested in HAMS and can therefore play a role in reducing racial/ethnic disparities in HAMS. For that reason, participating in screening may yield a sense of satisfaction from having engaged in an altruistic activity. Further, screening can improve a patient’s knowledge about HIV and HAMS, and also provides PLHA of color with access to the CTRU for the future when interest in HAMS and/or need for HAMS may be greater. Yet despite the importance of HAMS screening, little is known about participants’ screening experiences. However, such knowledge is vital and can be used to inform intervention efforts to tailor or improve the screening experience for under-represented groups.

### Aim of the present study

The overall goal of the present study is to describe the HAMS screening experience among PLHA of color, using both quantitative and qualitative data. We also explore potential gender and racial/ethnic differences in screening experiences, as well as potential differences based on HAMS eligibility. To explore factors underlying the decision to be screened for HAMS, as well as the experience of screening and the real-life context in which it occurs [25], we selected a modest number of descriptive case studies (N=3) to contextualize and enrich the quantitative findings.

## Methods

The present study focuses on a subset of participants enrolled in the cluster randomized controlled trial to evaluate the ACT2 intervention [10]. Participants in the present study are individuals enrolled in the study’s intervention arm who initiated screening by the time of the final follow-up interview (186 of 351 intervention arm participants). The larger study’s procedures are described in more detail elsewhere [10,9]. The study procedures were approved by the Institutional Review Boards at New York University and the partner CTRU.

### Quantitative assessments

Participants were assessed at three time points: baseline, and 4- and 12-months post-baseline, and received $25 for each. Interviews consisted of structured instruments, lasted approximately one hour, and were administered on laptop computers using both computer-assisted personal interviewing (CAPI) for the introductory sections and audio computer-assisted self-interviewing (ACASI) for the remainder of the interview at a project field site.

### Case study selection

In addition to structured interviews, a total of 35 in-depth qualitative interviews were conducted with a randomly selected subset of participants who were screened for HAMS from the intervention arm. For the present study, transcripts from these 35 interviews were placed into a random order. Starting with the first transcript, we examined whether the qualitative transcripts were generally similar to or different from the quantitative findings with respect to screening experiences. We examined transcripts until data “saturation” was reached, meaning that no new information or themes were observed in the data [26]. We reached saturation after reading 15 out of 35 transcripts, and no transcripts deviated significantly from the quantitative data, although there was variability in participants’ motives for screening and experiences during the encounter. We then purposively selected three cases from the 15 transcripts to illustrate the quantitative findings. All names used below in the case studies are pseudonyms, and other identifying details have been changed to protect participants’ confidentiality.

### Measures

#### Socio-demographic and health characteristics

At baseline demographic and background, characteristics such as gender, race/ethnicity, sexual orientation, and age were assessed with a structured measure [10]. Health indices, including date of HIV diagnosis, AIDS diagnosis, hepatitis C virus (HCV) diagnosis, CD4+ and viral load levels, and current or past use of antiretroviral therapy (ART), were assessed with items from the HIV Cost and Services Utilization Study [27]. Drug use, including lifetime injection drug use, was assessed with the Risk Behavior Assessment (RBA) [28].

#### Experiences of screening

At each follow-up interview, experiences of screening (14 items) were assessed using a modified version of the Experiences of Screening subscale of the Harris Survey [29,30]. The 14-item scale showed satisfactory internal consistency (Cronbach's α = 0.72). The 14 items consisted of a variety of Likert-type and yes/no scales, depending on the question, as we describe below. Quality of care received during screening was assessed on a 4-point scale (poor, only fair, good and excellent). Data were re-coded to indicate the percentage that reported good or excellent care during screening. The following experiences and attitudes were assessed with yes/no items: felt treated with dignity and respect, not subjected to more procedures and tests than necessary, did not feel treated like a guinea pig, was given enough time to talk to the person conducting the interview, felt questions were answered to satisfaction, would recommend a close friend living with HIV/AIDS to get screened for an HAMS at the site where they last got screened, and would recommend a family member get screened for an HAMS at the site where they last got screened. Two domains were assessed on a 5-point scale (not at all, a little, somewhat, quite a bit, a great deal): how comfortable they felt with the person conducting screening and how well they felt they could trust the person conducting the screening. These items were re-coded to indicate the percentage answering “a great deal.” The following domains were assessed on a 4-point scale (not well at all, not very well, somewhat well, very well): how well did they understand the screening process, how well did they feel they understood what HAMS are trying to study and how well they understood what they had to do if they joined an HAMS. These items were re-coded to indicate the percentage answering “very well.” Last, willingness to be screened again for a HAMS was assessed on a 4-point scale (not willing at all, not very willing, somewhat willing, very willing) and re-coded to indicate the percentage answering “very willing.”

### Missing data

A total of 198 individuals in the intervention arm initiated screening for HAMS. A total of 93.9% of these participants (N=186) provided data on their screening experiences. The most common reason for missing data on the Experiences of Screening scale was that participants did not report being screened in the interview, although they had presented for screening at the partner CTRU. This unexpected finding may also be due to the generic nature of the term “screening,” which is often confusing to people, because they are screened for so many conditions in a variety of settings [10]. In other cases, participants did not receive a follow up interview after their screening experience, and therefore could not report on their screening experience. Nonetheless, the majority of those screened provided data on their experiences, and these 186 individuals are the focus of the present study.

## Quantitative Data Analysis

Descriptive statistics (mean and standard deviation or percent) summarized demographic and health characteristics as well as the experiences of screening. The associations between race/ethnicity (African-American/Black vs. Latino/Hispanic), gender (male vs. female), eligibility (yes/no) and experiences of screening were examined using Fisher's exact test, with version 12 of Stata [31], since many of the contingency tables had cells with expected sizes of fewer than five cases.

## Results

### Quantitative

#### Characteristics of participants who presented for screening (N=186)

In Table 1 we provide a detailed description of the sociodemographic, health, and substance use characteristics of study participants, and highlight in this section some salient factors. Participants were aged 49.7 years old on average (SD = 7.4 years). More than a third was female (44.1%). Most were either African-American/Black (65.6%) or Latino/Hispanic (25.3%). Most (73.7%) identified as heterosexual. At the time of enrollment, about two-thirds (67.7%) were taking antiretroviral therapy (ART) and most (67.2%) reported an undetectable viral load. Only about a third (32.6%) had a CD4+ count of less than 350 per m/L. A majority (84.7%) had been diagnosed with HIV for over 10 years. About a third (31.7%) had been screened for an HAMS at some time in the past. A past history of alcohol and/or drug problems was common, but current high frequency alcohol and drug use, that is, at least weekly or more, was uncommon.

#### Experiences during screening

As presented in Table 2, participants’ experiences of screening were generally very positive. Most (96.2%) rated the quality of care they were given during screening as good-to-excellent. Almost all (98.9%) reported they did not feel they were treated as a “guinea pig” during screening and that they were given enough time to talk with the person who conducted the screening (97.9%). Further, almost all (98.9%) felt their questions were answered satisfactorily during screening. The majority (78.0%) felt a great deal of comfort with the person who conducted the screening.

#### Understanding screening and HAMS

Most (86.6%) participants reported they understood the screening process very well. Similarly, most (81.2%) stated they felt they understood what HAMS are usually trying to study, and what they would have to if they joined an HAMS (84.4%).

#### Future screening

Most (80.1%) stated that they were very willing to get screened for an HAMS again in the future. Almost all (98.4%) would recommend a close friend living with HIV/AIDS to get screened at the site where they got screened, and 96.2% would give the same recommendation to a family member.

#### Race/ethnic, gender, and study eligibility differences

There were no race/ethnic, gender, or study eligibility differences in experiences of screening.

## Qualitative

The three case studies are presented below, selected to complement the quantitative findings. While each case study participant necessarily had largely positive comments about the screening experience, each also had his or her own unique perspective on motivations for screening and the screening experience, including aspects of the ACT2 intervention as well as other factors.

### Case Study 1

Marc (male; age 46 years; infected with HIV for over 25 years; unemployed; engaged in regular health care; receives housing entitlements; currently taking ART; had not been screened for HAMS in the past)

#### Experiences with the ACT2 intervention

Marc credited the ACT2 intervention with broadening his perspective on HAMS. Marc, in general, valued gaining knowledge about HIV infection and health in general, and felt that the ACT2 intervention was consistent with that value. He described the group sessions as, *“very interesting and knowledgeable.”* Marc pointed out that while he was indeed given information about HAMS during the intervention, *“nothing was forced on (him),”* neither information nor participation in HAMS. He described the ACT2 intervention approach as making the concept of HAMS feel “*less threatening,”* and this got him *“more intrigued”* about what participating in HAMS could mean for him. He also reported coming into the intervention with a range of misconceptions and fears about HAMS, stating, *“(I had that) mentality …when you say ‘guinea pig.’ No, hell no, I’m not being nobody’s guinea pig, you know what I mean?”* However, Marc found that the more he discussed HAMS in the group and in the peer education session he conducted, the more comfortable he felt with the concept of exploring biomedical studies. Even the language he learned in the group sessions had an effect on his thinking. Marc noted, *“By me using the term, ‘clinical trial’ (as opposed to ‘experiments’), it’s kind a softening things up a little bit and it’s more interesting (to me).”*

#### Decision to be screened and experiences with screening

Regarding his reasons for deciding to be screened for HAMS, he reported: *“What I don’t know can’t hurt me, but the more I know can help me, you know what I mean? So by me getting screened it actually, to me it just made me feel better like knowing more about what I’m gonna do, the disease I’m battling with, you know what I mean? Because you can never know too much, but you can always know to less, which is no good, you know.”* He also noted that a desire to contribute to society by participating in biomedical research was another factor motivating his being screened for HAMS. Marc was not found eligible for a trial at the time of the screening, as is common in HAMS. Marc reported that he was not particularly disappointed about being found ineligible, saying he was not sure he was actually ready to participate in an HAMS at that point in time, but that he would consider enrolling in HAMS in the future.

Yet screening yielded an unexpected benefit for Marc: During the screening visit, he learned that the hospital where the CTRU was located also housed a large and well-regarded HIV clinic. Marc had long been unsatisfied with his health care provider and decided to switch to this clinic, in large part because he felt comfortable with the HAMS screening experience and the CTRU setting generally, which he experienced as professional, competent, and caring. He described his experience with changing health care settings as follows, *“I have a health care provider now (at the new clinic) and everything is good… And what was so cool about it is like when I was going to the clinical trial (screening), I’m like, the clinic is right here, and the clinical trials is right here, so you couldn’t give me no better, you know what I’m saying?... He’s (the doctor) got a beautiful clinic over there, I mean the staff is caring, they respect (me) and they are concerned.”* He was pleased that the HAMS screening location was now in the same building as his new primary health care provider, which, for him, reduced one of many structural barriers to screening that PLHA of color experience, as described above. Indeed, Marc stated his intention to screen for HAMS on a regular basis.

### Case Study 2

Susan (female; age 51 years; infected with HIV for over 10 years; currently in poor health; currently taking ART; and had not been screened for HAMS in the past)

#### Experiences with the ACT2 intervention

Susan has been HIV-positive for many years and suffered from many of the effects of HIV infection, including fatigue, memory loss, and neuropathy and also side effects from ART, such as diarrhea. She had also experienced a heart attack and a stroke in the past, leaving her quite frail. Further, Susan lived a life she described as *“hectic,”* because her grandson lived with her and she also took care of her granddaughter while her daughter was at work during the week. She described herself as very tired all the time and that her body felt older than it really was. Susan reported having a positive response to the intervention sessions. She noted, *“(HIV is) my problem, but it’s a nationwide problem…and you know, every time a new medication comes out, this is how it comes out (through HAMS)… and (it’s important to) to realize that we are at a place where we are today because other people did (HAMS).”* She continued to say that not having knowledge of HIV “*makes the disease 10 times worse,”* but at the same time, knowledge can be a burden. She herself struggled with how much information she wanted about HIV, but came to the conclusion that she would rather know everything there is to know.

#### Decision to be screened and experiences with screening

This desire for more knowledge about HIV influenced Susan’s decision to be screened, despite her doubts and fears about HAMS. On her feelings before the screening she said, *“I stress over the medication. That’s the only fear I really have, you know, but the good part is, is that I’m gonna be part of trying to make it better for the next person because that is my goal because I know what I go through with this.”* Susan reported that the staff at the CTRU was very helpful when she was screened. She felt welcomed there and that they took their time to explain the process to her and to answer all of her questions. She said, *“I meet him (the screener), I’m like wow, okay. We talked in depth, went through my (medical) history and he was just like so cool, and he get to the point and I had a couple of concerns.”* Susan was found eligible for an observational study, and gave her first blood sample that same day.

### Case Study 3

Juanita (female; age 35 years; infected with HIV for over 10 years; former heavy substance user; currently taking ART; and had not been screened for HAMS in the past)

#### Experiences with the ACT2 intervention

Juanita had been infected with HIV for over a decade at the time she joined the study. After initially learning she was infected with HIV, Juanita felt a desire to contribute to her community to help stop the spread of HIV to others. Juanita was struck by the fact that women commonly avoid HIV testing due to fears of stigma. She believed that education is the most important tool for HIV prevention, and it was this desire to learn more about HIV that motivated her to join the ACT2 Project. In the group sessions Juanita was struck by the large number of misconceptions about HAMS that are common in communities of color, and a high level of mistrust of HAMS, both of which she herself also shared. For example, she stated, *“I didn’t wanna be anyone’s guinea pig. (But) I had misconceptions. I always thought (a clinical trial) it was a pill and they gotta hook you up to all kinds of machines you know … A lotta times people think that you need to stop taking your medication in order to be in a trial and that’s not so, you know…. A lotta people think you have to give up your doctor. But you don’t.”* Further, Juanita was impressed by coming to understand that, if she enrolled in an HAMS, she would have a choice about continuing to participate, and she could drop out of a study at any time. She noted, *“It’s not like signing away your life (if you join an HAMS).”*

One component of the ACT2 intervention entails participants being trained how to educate their peers about HAMS. Grounded in her concern for her community and for vulnerable women in particular, Juanita particularly appreciated these aspects of the program. She found that during the course of conducting peer education, her own past misconceptions about and other negative attitudes toward HAMS continued to diminish. She noted, *“(ACT2) is (you) tell somebody and they tell somebody and the word gets around so then it’s more lax (relaxed), - you know, (after educating peers you are more) willing to be part of a study and be willing to be part of a trial.”*

#### Decision to be screened and experiences with screening

Juanita was screened for HAMS during the project and described her experience as quite positive. She noted that one of the most important aspects of screening was how she was initially welcomed at the CTRU. She said, *“So you know it all has to do with the approach of the (person who conducted the screening), and he was very nice, he was you know, friendly and open. It seemed like I had known him for you know, a lifetime. Everybody (at the CTRU) is like, real open, real friendly, real nice, nonjudgmental, and that sort a thing attracts people.”* Juanita was not found eligible for an HAMS at the time of screening, a fact that made her *“a little sad”* because she had a strong desire to contribute to research and to her community. However, she stated she planned to stay in touch with the CTRU to inquire about new studies as they open up, and the unit agreed to contact her when HAMS open up for which she might be eligible.

## Discussion

This is the first study to explore the experience of screening for HAMS among PLHA of color. Screening, whether done formally (as in the present study) or informally, is a crucial early step in the process of enrolling in HAMS. Yet PLHA of color are less likely to gain access to screening than their White peers. However, to date the experience of screening for HAMS has rarely been the focus of study, a gap the present study addresses.

Overall, participants had a robustly positive experience during the screening encounter, for example, reporting they were treated with dignity and respect, were able to understand HAMS better after the screening encounter, and would be willing to screen again and recommend screening to others. Screening experiences were positive regardless of participant gender, race/ethnicity, and HAMS eligibility. The case studies presented suggest the screening encounter was more pleasant than expected for some, as PLHA of color commonly experience fears and misconceptions of the CTRU system.

All participants in the present study engaged in the multicomponent social/behavioral ACT2 intervention prior to screening, which we surmise is a major contributing factor to the positive screening experience, as highlighted in the case studies. The ACT2 intervention provided participants with an opportunity to explore individual, organizational, social, and structural barriers that PLHA of color experience to HAMS, and articulate their fears and concerns about HAMS with peers prior to screening [8,10,23,20]. Thus, participants were primed to engage in and benefit from the screening encounter.

### Areas for improvement in screening

Five of our questions measured in the quantitative data were answered with less than 85% satisfaction, suggesting ways of improving the screening experience. These areas touched on trust and comfort with the person who conducted the screening, understanding what HAMS are trying to study and what is required of participants, and willingness to get screened again. These areas warrant exploration in future research to further improve the screening experience.

### Limitations

The study may be subject to self-selection bias [32] in that participants themselves chose to get screened. Thus, it is possible the very high enthusiasm for screening reported in the study is higher than what would be found among PLHA of color in general.

### Implications

Study findings have important implications for reducing racial/ethnic disparities in HAMS. Providers often assume that PLHA of color are not interested in HAMS, and therefore do not refer them to CTRUs for screening [2,33,34]. However, the present study suggests screening experience can be a valuable, positive, and productive encounter for PLHA of color that most would be willing to do again and recommend to family and friends. The absence of gender, racial/ethnic and eligibility differences in experiences of screening lead us to believe the screening process is well tolerated and positively viewed by PLHA with different backgrounds and life experiences. With additional work to address issues of comfort, trust, and patient understanding of what HAMS are trying to study, the screening experience could be very positive for an even greater percentage of PLHA of color. Settings that conduct or refer patients to HAMS, such as CTRUs, community-based organizations, and HIV clinics have the potential to reduce or eliminate racial/ethnic disparities in HAMS by first offering all patients regular and repeated access to HAMS screening, regardless of their potential eligibility or perceived interest [35], while implementing social/behavioral programs such as the ACT2 intervention to build patients’ skill and motivation to screen for and enroll into HAMS.

## Figures and Tables

**Table 1:** Socio-demographic and health characteristics of ACT2 participants at baseline who presented for screening by the 52 week follow-up

	Total (n=186)
**Socio-demographic characteristics**	
Female	44.1 %
Age Mean (SD)	49.7 (7.4)
Age 18–40	10.8%
Age 41–50	41.9%
Age 51+	47.3%
*Race/ethnicity*	
African American	65.6%
Hispanic	25.3%
White/Asian/Multiracial	9.1%
*Sexuality*	
Homosexual	16.7%
Heterosexual	73.7%
Bisexual	8.6%
Other	1.1%
**Health characteristics (self report)**	
Current ART	67.7%
Past ART	5.4%
ARV Naive	26.9%
CD4 < 350†	32.6%
CD4 < 500†	56.4%
Undetectable Viral Load‡	67.2%
HIV Diagnosis >= 10 Years Ago□	84.7%
AIDS Diagnosis■	59.2%
Ever Hepatitis C	31.7%
Ever Hepatitis B	18.3%
Prior ACT Screening	31.7%
**Substance use lifetime**	
Lifetime Alcohol Problem	44.6%
Lifetime Drug Problem	61.8%
Lifetime Alcohol or Drug Problem	68.3%
Ever Injected Drugs	28.0%
**Substance use past 3 months**	
Any Alcohol Use	50.5%
Weekly Alcohol Use	31.7%
Daily Alcohol Use	8.1%
Any Drug Use	39.3%
Weekly Drug Use	26.9%
Daily Drug Use	9.1%
Injected Drugs	1.6%
Any Alcohol or Drug Use	60.2%

† Response missing for five participants.

‡ Response missing for nine participants.

□ Response missing for ten participants.

■ Response missing for two participants.

**Table 2:** Experiences of screening among those who presented for screening by the 52 week follow-up.

	Total (n=186)
**Experiences during screening:** Quality of care received rated as good-to-excellent	96.2%
Felt treated with dignity and respect	98.4%
Was not subjected to more tests/procedures than necessary	97.9%
Did not feel treated as a guinea pig during screening	98.9%
Was given enough time to talk with the person who conducted the screening	97.9%
Felt that questions were answered to satisfaction	98.9%
Felt a great deal comfortable with the person who conducted the screening	78.0%
Felt they could trust the person who conducted the screening a great deal	67.2%
**Understanding of HAMS:** Felt they understood the screening process very well	86.6%
Understood very well what HAMS are usually trying to study	81.2%
Understood very well what they have to do if they joined a clinical trial	84.4%
**Future enrollment and recommendations:** Very willing to get screened again for an HAMS	80.1%
Would recommend a close friend living with HIV to get screened for an HAMS at the site where they last got screened	98.4%
Would recommend a family member living with HIV to get screened for an HAMS where they last got screened†	96.2%

† Response missing for three participants.
